# First report on molecular basis of *potato leaf roll virus* (PLRV) aggravation by combined effect of tuber and prevailing aphid

**DOI:** 10.1186/s13104-020-05370-1

**Published:** 2020-11-10

**Authors:** Ravi Ranjan Kumar, Mohammad Ansar, Kumari Rajani, Jitesh Kumar, Tushar Ranjan

**Affiliations:** 1 Department of Molecular Biology and Genetic Engineering, Bihar Agricultural University, Sabour, Bhagalpur, 813 210 India; 2 Department of Plant Pathology, Bihar Agricultural University, Sabour, Bhagalpur, 813 210 India; 3 Department of Seed Science and Technology, Bihar Agricultural University, Sabour, Bhagalpur, 813 210 India

**Keywords:** Potato leaf roll virus (PLRV), Tuber, Reservoir, Aphid, Virus transmission

## Abstract

**Objective:**

The *Potato Leaf Roll Virus* (PLRV) is one of the most devastating virus causing severe yield losses worldwide in potato. The comprehensive observations were made to study the PLRV infestation in major potato growing areas of Bihar (India) and further detailed molecular basis of PLRV aggravation was established.

**Results:**

Although aphids population were found comparatively lower with maximum symptomatic plants, our molecular data further confirms the presence of PLRV in all possible symptomatic tissues such as tubers, shoots and leaves. For the first time, we have proposed molecular basis of aggravation of PLRV, where tuber acts as a reservoir during off-season and further transmitted by aphids.

## Introduction

The potato crop is severely affected by various biotic stresses and among which viruses play a significant contribution in terms of huge loss in crop yield worldwide including India. Potato is affected by deadly viruses especially more potential infection via seed tubers due to the vegetative reproduction of the crop. More than 40 viruses and viroids hamper the cultivation of potato across the globe [1, 2]. The crop is infected by more than 30 RNA viruses, out of which 13 are mainly transmitted by aphids. Potato leaf roll virus (PLRV), belongs to genus *Polerovirus* and family *Luteoviridae*, is a widely spread potato virus worldwide and responsible for more than 20 million tonnes yield loss (up to 90%) globally [3]. PLRV is the only transmitted by aphids, namely, *Myzus persicae*. It is widely multiplied in the phloem tissue and the symptoms of disease reflect this position [4]. Because potato is a vegetatively propagated crop, once it gets infected with viruses, they can easily disseminate in the progeny tubers. These viruses are found in single or most of the time as a mixed infection within the potato crops. Tubers used for planting in next season can harbor latent viruses that subsequently reduce emergence, plant vigor and yield. All daughter tubers produced by infected mother tubers (secondary infection) will also get infected via systemic translocation of the virus during growth [5, 6]. Although, potato leaf roll disease is widespread throughout India but it is extremely critical in eastern plain zone, where population of vectors are very high in mid-February and March. In subsequent months, the higher temperature does not support the survival and growth of the aphids, makes them unavailable for potato host plants which ultimately breaks the leaf roll disease cycle. But in the subsequent season, infected tubers may provide primary inoculum which further spread aphids and thus affect the crop severely.

## Main text

### Disease surveys and specimen collection

During last decade, its incidence has also been observed in Bihar state, however, the severity was not reported earlier. During a field survey at nine districts of four agro-climatic zones of Bihar, plants exhibiting typical leaf roll symptoms were arbitrarily selected and examined for the possible causes. Intriguingly, we visited farmer plots of major potato producing districts of Bihar (Zone I-Samastipur, Vaishali and Muzaffarpur; Zone II-Purnea; Zone IIIA-Sheikhpura, Nawada, Nalanda, Patna; Zone IIIB-Bhagalpur, Banka) in the month of December-January for four consecutive years (2016 to 2019). The aphid population was found very low and at the same time the incidence of PLRV was found higher i.e. up to 25%. During the second survey in mid-February to March, the population of aphids were found higher (~ 10/plant). This data reflects the severity of this disease in eastern plain zone of India before multiplication of aphid populations. The plants exhibiting typical leaf roll symptoms, chlorosis and stunting were arbitrarily selected and further examined.

### Serological screening for infecting PLRV in potato plant leaves

Indirect enzyme linked immunoassay (ELISA) was performed for PLRV screening as described previously by Hobbs et al. [7]. Symptomatic and control leaves were macerated in 5 ml extraction buffer containing 0.05 M phosphate- buffer saline, 0.01 M sodium diethylene carbamide at pH 7.4. The extracts were centrifuged at 10,000*g* for 5 min. ELISA plate was incubated overnight at 4 °C with 100 μl of supernatant. The plate was washed and blocked with skim-milk powder. Two hundred microlitre of rabbit polyclonal antibodies against PLRV-CP (Promega) (diluted 1:500 in PBS) was added to each well and incubated for 3 h at 37 °C. After washing, secondary antibody goat anti-rabbit immunoglobulin G conjugated with alkaline phosphatase (Promega) diluted 1:1000 was added to the wells and incubated for 3 h at 37 °C. The plate was developed with the substrate p-nitrophenyl phosphate (p-NPP) and OD at A405 nm was measured.

### RT-PCR analysis of viral RNA in potato

Based on the results obtained by Indirect-ELISA, the total RNA was extracted from symptomatic leaves of potato using RNeasy Plant Mini kit (Thermo Scientific) following the manufacturer’s instructions. RT-PCR was carried out using PLRV_CP gene specific primers (FP: 5′-ATGAGTACGGTCGTGGTTAGAGG-3′; RP: 5′-CTATCTGGGGTTCTGCAAAGCCAC-3′). cDNA synthesis was carried out using RT-PCR kit (Thermo Scientific). Total reaction volume of 20 μl contained RNA template, sterile water, 10 mM dNTPs mix and 50 μM random nanomers. Initially, the reagents were incubated at 70 °C for 10 min, later on, sterile water, 10 × RT-buffer, 20 U/µl RNase inhibitor and 20 U/µl enhanced AMV-RT were added and incubated at 25 °C for 15 min and 45 °C for 50 min. PCR was performed keeping the total reaction volume as 50 μl. Further, TA cloning approach was employed to sequence all the amplified *cp* gene from different parts of infected plants. Sequence of *cp* gene was further submitted to GenBank to get the accession number (MW027216). The details of submitted sequence are given in the “availability of data and materials” section.

### Results

**Fig. 1:**
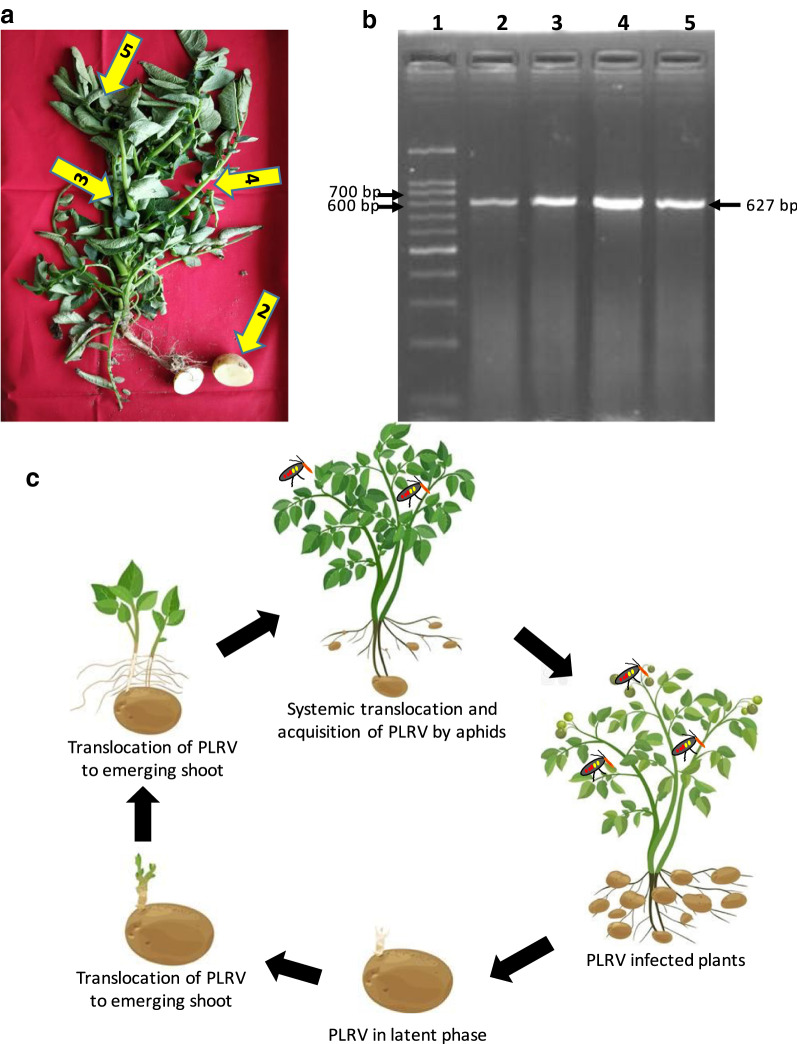
**a** Infected PLRV Plant sample. **b** Detection of PLRV in different parts of infected potato plant using CP specific primer (bp). Lane 1: DNA ladder; Lane 2: tuber; Lane 3: main shoot; Lane 4: branched shoot and Lane 5: leaf **c** Model for transmission of PLRV by collective influence of potato tuber and aphids (potato cartoons were adopted from dreamstime.com)

The infected samples were first tested through DAS-ELISA by following the standard protocol. Further from the positive PLRV samples identified through DAS-ELISA, total RNA was extracted from mid rib and major veins of infected leaf using RNA extraction kit and first strand cDNA was synthesized using cDNA synthesis kit (Fermentas) with respective primers. Finally, to confirm the PLRV infection, RT-PCR was performed using PLRV coat protein gene specific primers. Total RNA were extracted from all possible parts of infected plant (viz*.,* tuber, main shoot, branched shoot and leaf) and electrophoresis analysis of RT-PCR product showed a single amplified fragment of 627 bp (Fig. 1a, b). Further, TA cloning approach was employed to sequence all the amplified *cp* gene from different parts of infected plants.

We used same tubers from infected plant as a seed for next season cropping in controlled environment and all the results were further validated. Our consistent data of four cropping seasons indicate that these tubers act as a reservoir for these viruses and leads to the emergence of unhealthy progeny plants. From infected tubers, all the parts of progeny plants could get infected via systemic translocation of the PLRV (Fig. 1c). Because the aphid population increases exponentially during late winter season, the probability that they will encounter an infected plant is higher. Aphids acquire the PLRV into the salivary glands by feeding on infected plants and further continue to transmit the virus to healthy plants for its remaining life and completes the disease cycle (Fig. 1c). Our present study also supports the findings of Leonard and Holbrook on role of aphid in PLRV pathogenicity. Control depends solely on the use of disease-free seeds for planting [8, 9]. Farmers of indo-gangetic plain of this region import the potato seeds from neighbour states or local companies, where risk of getting unhealthy seeds is very high. As a result, the pressure of production of disease-free seed potato has become a major challenge. The most effective approach to manage the viral diseases is planting of healthy and virus free seed potato. The diagnosis and management of aphids are considered to be an important step towards preventing spread of the viruses in potato. Our lab is also trying to the development of a rapid, reliable and cost-effective protocol to detect simultaneously multiple virus infection in a single assay in plants and tubers (Funded by DBT, Govt. of India).

Although, the study of Wright and Bishop [6] reveals that potato tubers may act as volunteer source for PLRV and potato virus X, but the molecular basis for PLRV aggravation has been not reported so far. Our present study has dissected the mechanism of transmission of PLRV by collective influence of potato tuber and aphids. As PLRV has emerged as a devastating disease in the eastern indo-gangetic plain during the last 5 years, the small and marginal farmers of this region are suffering with huge economical loss due to inadequate screening of quality of tubers. Till date, there was no reports on how PLRV spreads and affect the crop production particularly in this region, the present study validates the findings of disease biology of PLRV in order to find out an effective and timely management. Our present findings have opened up an opportunity for developing strategies for timely management of PLRV before planting and/or before rising up the aphid populations by breaking out the chain in the peak season of aphid multiplication. Our lab is also involved in the development of kit based diagnosis of tubers for early identification and further conducting knockdown study of PLRV, which could help the scientific community in order to undertake long term management against this disease. This work will also have a significant impact on scientific community for developing PLRV resistance plant *vis-a-vis* agricultural sector to produce disease-free potato seed.

## Limitations

- Despite all efforts so far, the detailed molecular mechanism of PLRV aggravation is poorly understood.
- Development of field based viral diagnostic kit and PLRV resistance plant are the best way to produce disease-free potato seed.

## Data Availability

GenBank accession number of *cp* gene: MW027216. ATGAGTACGGTCGTGGTTAAAGGAAATGTCAATGGTGGTGTTCAACAACCAAGAAGGCGAAGAAGGCAATCCCTTCGCAGGCGCGCTAACAGAGTTCAGCCAGTGGTTATGGTCACGGCCCCTGGGCAACCCAGGCGCCGAAGACGCAGAAGAGGAAGCAATCGCCGCTCAAGAAGAACTGGAGTTCCCCGAGGACGAGGCTCAAGCGAGACATTCGTGTTTACAAAGGACAACCTCATGGGCAACTCCCAAGGAAGTTTCACCTTCGGGCCGAGTCTATCAGACTGTCCGGCATTCAAGGATGGAATACTCAAGGCCTACCATGAGTATAAGATCACAAGCATCTTACTTCAGTTCGTCAGCGAGGCCTCTTCCACCTCCTCCGGTTCCATCGCTTATGAGTTTGGCCCCATGTGCAAAGTATCATCCCTCCGGTCCTACGTCAACAAGTTCCAAATTACGAAGGGCGGCGCCAAAACTTATCAAGCGCGGATGATAAACGGGGTAGAATGGCACGATTCTTCTGAGGATCAGTGCCGGATACTGTGGAAGGGAAATGGAAAATCTTCAGATACCGCAGGATCCTTCAGAGTCACCATCAGGGGGTGGCTTTGCAAAACCCCAAATAG.

## Abbreviations

PLRV: Potato leaf roll virus
CP: Capsid protein
RT-PCR: Reverse transcriptase-polymerase chain reaction
